# Identifying Key Biomarkers in Pediatric Pulmonary Hypertension: An Investigative Approach

**DOI:** 10.3390/children11060737

**Published:** 2024-06-17

**Authors:** Farida Mindubayeva, Lyudmila Akhmaltdinova, Mariya Ospanova, Bibigul Tukbekova, Zhanat Bolatbekuly, Yuliya Niyazova, Yelena Salikhova, Olga Avdienko, Meruert Akhmetova

**Affiliations:** 1 Department of Physiology, NCJSC «Karaganda Medical University», Karaganda 100000, Kazakhstan; mindubaeva@qmu.kz (F.M.); niyazovay@qmu.kz (Y.N.); salihova@qmu.kz (Y.S.); ahmetovam@qmu.kz (M.A.); 2 Scientific Research Laboratory, NCJSC «Karaganda Medical University», Karaganda 100000, Kazakhstanavdienko@qmu.kz (O.A.); 3 Department of Pediatrics and Neonatology, NCJSC «Karaganda Medical University», Karaganda 100000, Kazakhstan; tukbekova@qmu.kz; 4 Municipal State Enterprise «Multiprofile Hospital No. 2 of Karaganda», Health Department of the Karaganda Region, Karaganda 100000, Kazakhstan; zhanat_b_78@mail.ru

**Keywords:** 5-hydroxyindoleacetic acid, children, congenital heart defects, insulin-like growth factor 1, pulmonary hypertension

## Abstract

This study assesses the utility of early biomarkers—5-hydroxyindoleacetic acid (5-HIAA) and insulin-like growth factor 1 (IGF-1)—for diagnosing and monitoring pulmonary hypertension (PH) in children with congenital heart defects (CHD). Due to the risks associated with invasive diagnostics, such as right heart catheterization, non-invasive biomarkers provide a safer alternative for early PH detection. This cohort-based study utilized blood and urine samples to measure 5-HIAA and IGF-1 levels via enzyme immunoassays. Our findings revealed significant changes in 5-HIAA concentrations across various biological matrices, supporting its potential as a diagnostic tool. Specifically, altered levels in urine and plasma reflect its role in serotonin metabolism and vascular remodeling in PH. IGF-1 levels were notably reduced in plasma, suggesting its involvement in PH pathophysiology. ROC analysis confirmed the diagnostic efficacy of these biomarkers, particularly 5-HIAA’s high specificity and sensitivity. In conclusion, 5-HIAA and IGF-1 levels correlate well with PH, underscoring their diagnostic value for early PH detection in children with CHD.

## 1. Introduction

Pulmonary hypertension (PH) in children is a significant clinical challenge, especially when it is associated with congenital heart defects (CHD). This condition is characterized by increased pressure in the pulmonary artery, it significantly worsens the prognosis of treatment for children with CHD and increases not only the risk of serious complications, but also mortality. The current difficulties with the diagnosis of PH are due to the common symptoms and limited capabilities of standard procedures and emphasize the need to develop more accurate and safe diagnostic methods [1,2,3].

The non-specificity of PH symptoms, such as breath shortness, fatigue, and dizziness, makes early detection of the disease especially difficult. Moreover, invasive diagnostic procedures, for example, catheterization of the right heart, which is the gold standard for the diagnosis of hypertension, pose risks for pediatric patients. In this context, the study of biomarkers opens up new prospects for early and accurate diagnosis of PH [4].

The importance of the topic of studying PH in children cannot be overestimated in modern medicine. This condition not only affects the quality of life of young patients and their families, but also poses significant challenges to the healthcare system as a whole. Early detection and adequate treatment can significantly improve the prognosis of the disease, reduce the risk of complications and increase the chances of children living a full life [5,6,7].

The development and validation of biomarkers for PH can radically change approaches to the diagnosis and treatment of this condition. Biomarkers reflecting changes in the structure and function of pulmonary vessels, endothelial dysfunction and remodeling processes in the heart muscle can provide early detection of PH even before the first clinical signs appear [8].

Thus, current research in the field of PH biomarkers represents a promising direction in pediatric cardiology and pulmonology. The results of such studies not only contribute to improving diagnostic capabilities but also open up new horizons for the development of individualized therapeutic strategies based on the molecular mechanisms of disease development. Continued research in this area remains a priority for scientists and clinicians around the world seeking to ensure a better future for children with PH [9].

The objective of our research was to investigate the role and potential of early biomarkers, specifically the serotonin system and insulin-like growth factor 1, in the diagnosis, monitoring, and prognosis of pulmonary hypertension in pediatric patients with congenital heart defects.

## 2. Materials and Methods

The study was cohort-based by design. The permission for the study was received from the Ethical Committee of Karaganda Medical University No. 37, dated 29 March 2022. The parents of all patients included in the study signed an informed consent for the research. The recruitment of participants was carried out at the Center of Cardiac Surgery in Karaganda city (Kazakhstan) in the period from June 2022 to December 2023. The children were divided into two groups. Group I consisted of children with CHD complicated by PH. Group II (control group) included healthy children without CHD and PH. The criteria for inclusion in group I included the following parameters: the presence of a diagnosed congenital heart defect in children, pulmonary hypertension, absence of concomitant infectious complications, age from 0 to 7 years, consent of parents/official representatives of children to participate in the study. The criteria for inclusion in group II were as follows: absence of severe somatic pathology, including CHD and PH, absence of active infectious and inflammatory processes, age from 0 to 7 years, consent of parents/official representatives of children to participate in the study.

Laboratory methods. In the context of this study, as experimental material, we used whole blood stabilized with citrate for platelet extraction and plasma treated with EDTA to produce the platelet-poor plasma (PPP), as well as a single morning urine portion. The selection of biological samples (blood and urine) was carried out before the start of surgery.

To produce platelet-rich plasma (PRP), the samples were centrifuged for 5 min at room temperature with a centrifuge force of 200 g. The supernatant was transferred to a separate tube for subsequent determination of the platelet count using a Mindray BC-3200 hematology analyzer. To isolate platelets, 1600 µL of isotonic solution was added to 400 µL of PRP and re-centrifuged at 4500 *g* and 4 °C for 10 min. The supernatant was removed, and 400 µL of distilled water was added to the platelet sediment, after which thorough mixing was carried out on a vortex. The resulting platelet suspension was stored frozen at a temperature of −70 °C. The concentration of analyte in platelets was calculated taking into account their number in the sample and expressed in picograms per 10^9^ platelets, based on the data obtained at the stage of their isolation.

To obtain PPP, the sample was centrifuged for 10 min at 2000 *g*. Then the plasma was distributed into aliquots and stored frozen at a temperature of −70 °C until analysis.

A single morning urine sample was used in the urine analysis. Urine aliquots were frozen at −40 °C before analysis. The concentration of analytes in urine was adjusted, taking into account the creatinine content determined in this portion, in order to standardize the dilution of the sample.

Enzyme immunoassay. ELISA kits manufactured by Cloud-Clone Corp were used to determine the level of serotonin, 5-hydroxyindoleacetic acid (5-HIAA), and insulin-like growth factor 1 (IGF-1), according to the manufacturer’s instructions. 

Statistical analysis. Statistical analysis was performed using GraphPad Prism for intergroup comparisons and Statistica (StatSoft 10.0) software for correlation analysis. The data did not follow a normal distribution, and to better mitigate noise and outliers in the small sample size, non-parametric methods were employed. The results in the tables are presented as medians, with the first and third quartiles indicated. Intergroup comparisons were conducted using the non-parametric Mann–Whitney test.

Spearman’s rank correlation method was used to assess the relationship between the variables. Only correlation coefficients with a *p*-value of less than 0.05 were considered in the analysis.

The primary goal was to evaluate the diagnostic power of the proposed indicators, for which classical ROC analysis (receiver operating characteristic) was conducted using the DATAtab online statistics calculator (datalab.net). Diagnostic characteristics of the tests were determined using the MedCalc Software Ltd version 22.026.

## 3. Results

During the study, we concentrated on analyzing the population of children with CHDs complicated by PH and control group. The inclusion of a control group allowed for a more precise determination of the relationship between the investigated molecular mechanisms and PH, potentially enhancing future diagnostic and therapeutic approaches for this condition. The characteristics of the patients are described in Table 1.

When studying the content of 5-HIAA in the experimental group (Table 2), we found a decrease in its concentration in plasma (*p* = 0.03), an increase in urine (*p* = 0.026), and no changes in platelets compared with the control group. 5-HIAA is a serotonin metabolite, which, according to the literature data, may be associated with vascular remodeling and vasoconstriction in the context of PH. Since serotonin metabolism is in a complex equilibrium between platelet depot, plasma concentration, and excretion of its metabolites in urine, we introduced indices that reflect this equilibrium. At the same time, the platelet/plasma 5-HIAA index was significantly increased in patients (*p* = 0.001), and the ratio of 5-HIAA between plasma and urine was even more significantly reduced (0.95 vs. 17.5, *p* = 0.007).

The level of IGF-1 in patients was significantly reduced in blood plasma (*p* = 0.001), its absolute median content in platelets did not change, however, together this led to the fact that the ratio between plasma and platelets shifted sharply almost tenfold (*p* = 0.001).

The Spearman correlation analysis was performed to determine the specificity of the shifts of studied PH markers Table 3.

According to the rank correlation data presented in the table, both positive and negative correlations were observed between metabolites and functional test data, such as the calculated pulmonary arterial systolic pressure (PASP) and the mean pressure gradient (PGmean), as well as between them.

To determine the diagnostic significance of these markers, ROC analysis was performed with the calculation of AUC, an empirical cutoff level and diagnostic characteristics.

**Figure 1:**
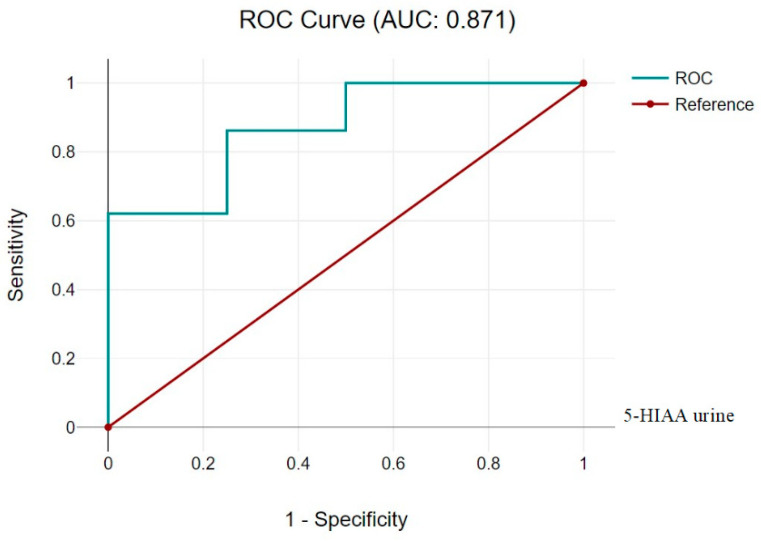
5-HIAA urine.

5-HIAA urine is a potential marker of PH, reflecting the metabolism of the serotonin system in the body. In our study, the AUC for 5-HIAA urine was 0.871 (95% CI 0.735, 1.005), with strong data on specificity and sensitivity, indicating high test accuracy. The positive predictive value was very high, which suggests good test precision when results are positive (Figure 1 and Table 4). Cutoff 5-HIAA urine was taken as 3 (ng/moles of creatinine).

It was not advisable to calculate the diagnostic significance for 5-HIAA plasma and 5-HIAA platelet, since there was no significant difference in platelet levels between the groups. The range of changes in the plasma case was small for the possibility of making a clinical decision on the level of the marker.

However, for the calculated indices of 5-HIAA metabolism between body environments, such a calculation has become possible.

**Figure 2:**
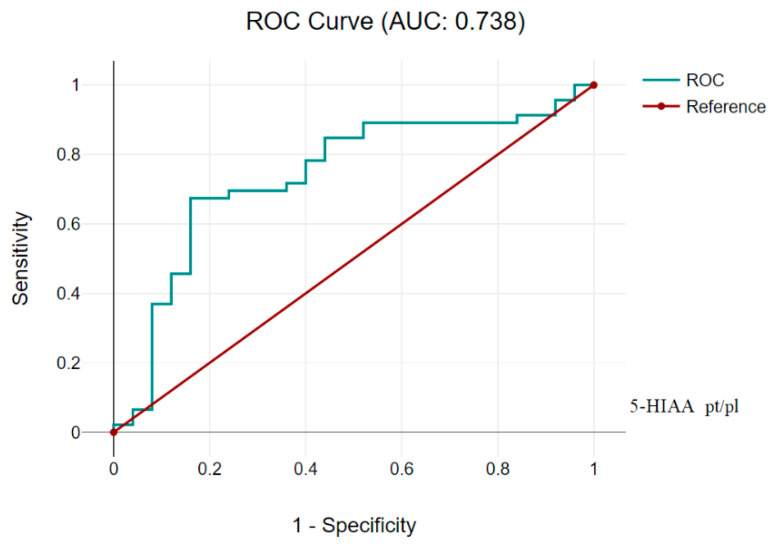
5-HIAA platelet/plasma AUC 0.738 (CI 95%, 0.623, 0.853).

Comparing the data for 5-HIAA platelet/plasma at Cutoff 1, 2 in the previous table, generally, we see similar characteristics, although the sensitivity of this test is lower, which may indicate a greater number of missed true positive cases (Figure 2 and Table 5).

5-HIAA plasma/urine demonstrated better sensitivity and accuracy (AUC 0.897 (CI 95% 0.781, 1.013)) at Cutoff 3.5.

**Figure 3:**
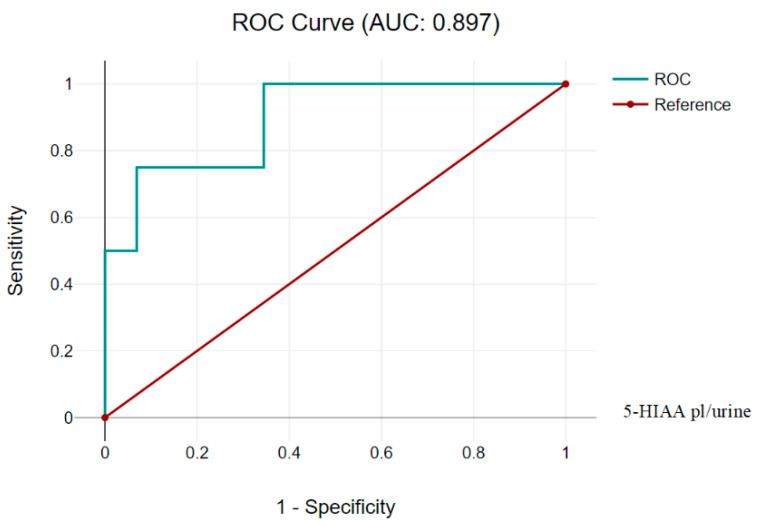
5-HIAA plasma/urine 0.897 (CI 95%, 0.781, 1.013).

The positive predictive value is almost identical to the previous 5-HIAA platelet/plasma test, but the negative predictive value is slightly lower, and it may be important when considering the use of this test for screening or confirmation diagnosis (Figure 3 and Table 6).

As a result, none of the new calculated indicators exceeded the benefits and significance of 5-HIAA in urine, but clearly exceeded it in terms of cost and complexity of analysis.

IGF-1 as a promising new marker of PH showed encouraging results when comparing groups. We tested its diagnostic significance to compare and understand whether it improved diagnostic results obtained with the commonly accepted 5-HIAA in urine.

**Figure 4:**
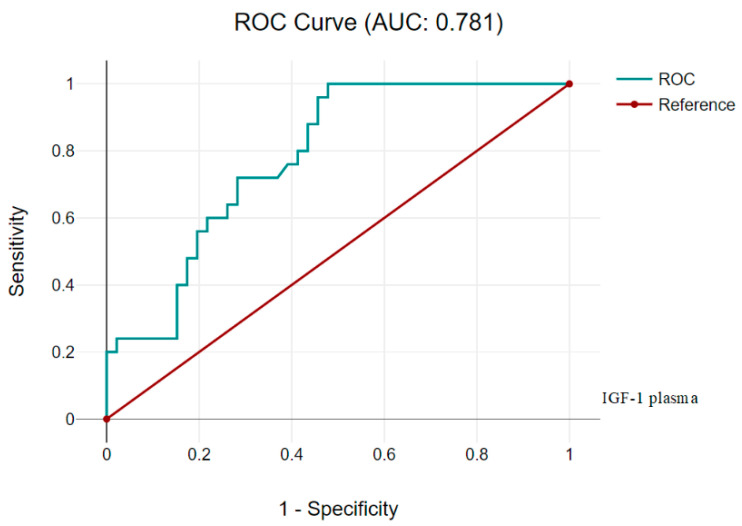
AUC for IGF-1 in plasma 0.781 (CI 95%, 0.675–0.887).

As a result, the ROC analysis showed excellent specificity for the IGF-1 plasma test, although low sensitivity was recorded, the AUC was 0.781 (CI 95%, 0.675–0.887) (Figure 4).

The diagnostic characteristics were as follows (Table 7) when using Cutoff IGF-1 in plasma at the level of 11 ng/mL. The positive prognostic value and specificity are impressively high, which makes this test a good tool for confirming a diagnosis.

**Figure 5:**
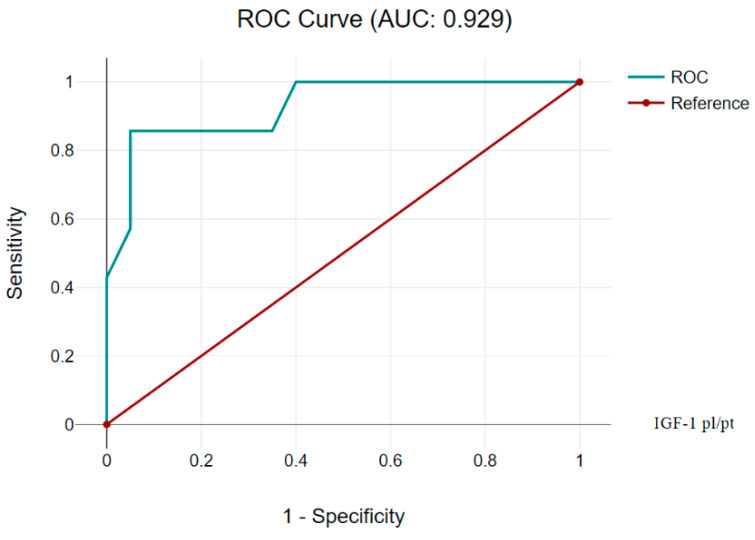
IGF-1 index (plasma/platelet) AUC 0.929 (CI 95%, 0.832–1.00).

On the contrary, the complex calculated IGF-1 test, expressed as the ratio of content in plasma to content in platelets, showed outstanding diagnostic characteristics of AUC 0.929 (CI 95%, 0.832–1.00) (Figure 5). This test is one of the most accurate of the considered tests, has a high sensitivity, and a high specificity, as well as high PPV and NPV values; this makes it a reliable diagnostic tool.

The diagnostic characteristics were as follows when using the Cutoff IGF-1 index (plasma/platelet) at the level of 0.65 (Table 8).

## 4. Discussion

We observed significant differences in the levels of 5-HIAA and IGF-1 between the PH group and the control group. Specifically, there was a significant decrease in plasma 5-HIAA (*p* = 0.03) and an increase in urinary 5-HIAA (*p* = 0.026) in the PH group, while platelet 5-HIAA levels remained unchanged. Moreover, IGF-1 levels were significantly reduced in the blood plasma of patients with PH (*p* = 0.001). Our data also revealed altered indices reflecting the equilibrium between plasma and platelet 5-HIAA and IGF-1, highlighting potential disruptions in serotonin and IGF-1 metabolism in PH patients.

Our study reveals that IGF-1 significantly influences the adaptation of the heart and vascular system to physiological requirements and stressors. We found that IGF-1 activates a series of intracellular signaling pathways through the IGF-1 receptor, promoting cell survival, proliferation, and improved metabolism. These findings align with previous studies by G. A. Aguirre and others, which highlight IGF-1′s role in improving cardiomyocyte survival, reducing apoptosis, and supporting cardiac recovery [10,11,12,13,14,15,16]. For example, in the context of myocardial infarction, our study confirms IGF-1’s protective properties, potentially limiting the size of a heart attack and preserving heart functionality, similar to the protective mechanisms detailed by Aguirre and Castilla-Cortazar [10].

In addition to its role in myocardial maintenance, IGF-1 extends its beneficial effects on vascular health. Our findings indicate that IGF-1 stimulates endothelial function and has anti-atherogenic properties, crucial for preventing the development and progression of atherosclerosis, the main cause of cardiovascular diseases. By enhancing nitric oxide production and modulating inflammatory processes in the vascular wall, IGF-1 plays an important role in vascular homeostasis, maintaining endothelial function, and preventing the early stages of atherosclerotic disease. These results corroborate the studies conducted by L. Espinosa and colleagues, who demonstrated IGF-1’s impact on endothelial function and cardiovascular disease mitigation [10].

The widespread effect of IGF-1 on the cardiovascular system positions it as a promising biomarker for cardiovascular diseases. Fluctuations in circulating IGF-1 levels correlated with various aspects of cardiovascular disease, including myocardial infarction, heart failure, and atherosclerosis, positioning IGF-1 as a potential prognostic marker. Although lower IGF-1 levels have been associated with an increased risk of cardiovascular events and mortality, indicating its potential in predicting cardiovascular risk, the relationship between IGF-1 concentrations and cardiovascular outcomes demonstrates a degree of complexity. This complexity highlights the need for further research to fully understand its potential as a clinical biomarker in cardiology [10,11,12,13,14,15,16].

While synthesizing the current understanding of the role of IGF-1 in cardiovascular health, it becomes evident that IGF-1 acts not only as a key regulatory molecule but also offers a promising pathway for therapeutic intervention and the development of biomarkers in the management of cardiovascular diseases. Its ability to promote cardiomyocyte survival, improve vascular health, and mitigate atherosclerotic processes determines the therapeutic possibilities inherent in targeting IGF-1 pathways. Ongoing research aimed at uncovering the complex interactions of IGF-1 and cardiovascular health promises to open new strategies to combat cardiovascular disease, harnessing the potential of IGF-1 to improve outcomes for cardiovascular patients and their care. As research on the effects of IGF-1 on the cardiovascular system continues, it foreshadows integration into the framework of personalized medicine, offering individualized therapeutic approaches based on IGF-1 profiles and cardiovascular risk assessments [10,11,12,13,14,15,16].

The relationship between serotonin, IGF-1, and pulmonary hypertension (PH) involves complex interactions that contribute to the pathogenesis and progression of the disease. Serotonin, as a neurotransmitter and vasoactive mediator, promotes the proliferation of pulmonary smooth muscle cells and vasoconstriction, key signs of PH. Its action is mediated through serotonin receptors and the serotonin transporter (SERT), which affect serotonin availability and signaling. Increased serotonin levels and altered signaling are associated with the development of PH, leading to vascular remodeling and increased pulmonary vascular resistance. IGF-1, acting through IGF-1 receptors, plays a role in cell growth, survival, and metabolism, exerting protective effects on the cardiovascular system, including support for endothelial function, inhibition of smooth muscle cell proliferation, and protection against oxidative stress and apoptosis. These IGF-1 actions may counteract some pathological processes in PH, such as vascular remodeling and endothelial dysfunction. The interaction between serotonin and IGF-1 signaling in PH is complex, with serotonin potentially indirectly influencing IGF-1 signaling through its effects on vascular tone and smooth muscle cell proliferation [10,11,12,13,14,15,16].

Studies conducted by G. A. Aguirre in 2010 and 2018 focus on the significant role of IGF-1 in metabolism regulation and cardiovascular system protection. Aguirre’s studies explore the mechanisms of action of IGF-1 within the growth hormone/IGF-1/insulin axis, highlighting its key role in managing metabolic processes that precede the development of cardiovascular diseases. Specifically, Aguirre focuses on the relationship between IGF-1 and mitochondrial protection, the improvement of metabolic processes in the cardiovascular system, and antioxidant properties in various organs.

G. A. Aguirre, along with González-Guerra, expands the study of the molecular and biochemical pathways of IGF-1, analyzing its signaling mechanisms in the heart and exploring both canonical and non-canonical pathways. Their work highlights the interaction of IGF-1 with cardiovascular tissues, detailing its effects on endothelial function and promoting anti-inflammatory processes in the cardiovascular system.

In 2018, L. Espinosa et al. studied the effects of IGF-1 receptors on the heart and the possibilities of modulating IGF-1 cellular pathways to mitigate cardiovascular disease, providing important data for developing new therapeutic strategies.

In 2010 and 2018, I. Castilla-Cortazar examined the association of IGF-1 deficiency with the development of cardiovascular diseases and cerebrovascular accidents, such as strokes. His research provides extensive data on the physiological role of IGF-1 in preventing oxidative and apoptotic/necrotic damage in the cardiovascular system and reveals the potential therapeutic use of IGF-1 in treating cardiovascular disorders [10].

Bouzina et al. identified a link between IGF-1 signaling and the development of tumor processes and found that high levels of IGF binding protein (IGFBP)-1 in the blood are associated with worse survival in pulmonary arterial hypertension (PAH). These findings highlight the potential role of IGF-1 in the pathogenesis of PAH and provide a basis for further research on modulating this pathway in the context of the disease [16].

Currently, 5-HIAA, as the main metabolite of serotonin, attracts the attention of scientists because of its characteristic pathophysiological involvement in the condition of pulmonary vessels. 5-HIAA accumulates as the end product of the metabolic breakdown of serotonin, a vasoactive mediator whose involvement in the pathogenesis of pulmonary artery remodeling is well known. Increased serotonin levels are associated with proliferative changes in the walls of the pulmonary arteries, which is a distinctive feature of PH. Thus, the quantification of 5-HIAA provides an indirect but meaningful look at serotonin turnover, potentially reflecting abnormal activity contributing to changes in pulmonary vessels. Recent studies have used enzyme immunoassay to measure levels of 5-HIAA in various biological matrices, including blood plasma and urine, comparing the results with echocardiographic markers of PH, such as tricuspid regurgitation and right ventricular dilation. The correlation of high levels of 5-HIAA with these parameters highlights its potential as a non-invasive biomarker of PH. As research progresses, the role of 5-HIAA as a biomarker of PH continues to be clarified, promising to become a reference point for new targeted therapeutic strategies. This integration of metabolic, molecular, and morphological aspects into the context of PH not only clarifies the diagnostic view but also expands the prognostic plan, potentially directing individualized patient management in this multifaceted disease [17,18,19,20,21,22,23].

Advantages of the study. This novel study explores the molecular and biochemical pathways of IGF-1 in the heart and its interaction with the serotonin system, offering valuable clinical insights.

Limitations of the study. The limitations of the study are the small sample size. IGF-1 is a non-specific marker that may generally reflect the severity of the child’s condition; however, the presence of correlations with PH data and correlations with the generally recognized diagnostic markers 5-HIAA in urine, to some extent, negates this limitation. We did not use daily urine to study the secretion of serotonin metabolites, but standardization for creatinine levels allowed us to improve the specificity of the study. We used RUO kits for research, not having access to IVD kits due to bureaucratic restrictions, which could also affect the accuracy of the research.

## 5. Conclusions

Our research highlights the significant roles of the serotonin system and IGF-1 in the development of pediatric pulmonary hypertension (PH). Our data show strong links between these substances and PH, suggesting their potential as biomarkers for diagnosis and prediction.

Serotonin regulates vascular tone and contributes to pathological processes in the pulmonary arteries, while IGF-1 influences the growth, development, and repair of vascular cells. Changes in these molecules’ levels may reflect PH mechanisms, making them promising for new diagnostic and treatment methods.

Despite our findings, further studies on larger patient populations are needed to confirm these roles and investigate their interactions with other molecules. We also propose a new method for assessing IGF-1 levels as a potential PH biomarker in children, which could enhance personalized therapies. However, more research is needed to confirm its clinical effectiveness.

In conclusion, our study advances the understanding of PH in children and opens new avenues for diagnosis and treatment. Significant challenges remain in further investigating serotonin and IGF-1 and developing effective methods based on these findings.

## Figures and Tables

**Table 1:** Baseline patient characteristics.

	I Group PH	II Group Health Control
Number of Children	46	24
Median Age (weeks)	8.5 [6.0; 48]	8.0 [6.0; 21]
CHD (%)	100%	Control
PH (%)	100%	Control
Ventricular septal defect (VSD) (%)	44%	Control
Atrial septal defect (ASD) (%)	42%	Control
VSD and ASD (%)	8.88%	Control
Patent ductus arteriosus (ODA) (%)	4.44%	Control

**Table 2:** The content of the main analytes in the studied groups.

Biomarkers	PH Me [Q1; Q3]	Health Control Me [Q1; Q3]	*p*-Level
5-HIAA plasma (ng/mL)	8.20 [6.79; 11.57]	11.33 [9.28; 12.50]	0.03
IGF-1 plasma (ng/mL)	9.21 [6.11; 16.00]	17.01 [13.05; 19.11]	0.0001
5-HIAA platelet (ng/10^9^)	11.10 [9.10; 14.01]	8.10 [7.02; 12.59]	0.17
IGF-1 platelet (pg/10^9^)	146.79 [100.60; 201.92]	83.96 [75.56; 104.04]	0.12
5-HIAA platelet/plasma	1.50 [1.34; 1.92]	0.99 [0.73; 1.19]	0.001
IGF-1 plasma/platelet	0.08 [0.05; 0.17]	1.19 [0.69; 1.24]	0.0012
5-HIAA urine (ng/moles of creatinine)	6.55 [3.25; 12.68]	1.00 [0.43; 2.48]	0.026
5-HIAA plasma/urine	0.95 [0.65; 3.3]	17.5 [4.6; 28.7]	0.007

**Table 3:** Spearman rank order correlations at *p* < 0.05.

1	2	Spearman Rank
IGF-1 plasma	PASP mmHg	−0.44
IGF-1 plasma	PG_mean_ in the trunk	0.42
IGF-1 plasma	5-HIAA plasma	0.22
IGF-1 plasma/platelet	PASP mmHg	−0.65
5-HIAA plasma	PASP mmHg	−0.42
5-HIAA urine	PASP mmHg	0.49
5-HIAA plasma/urine	IGF-1 plasma	0.29
5-HIAA plasma/urine	PASP mmHg	0.52
5-HIAA platelet/plasma	PASP mmHg	0.49

**Table 4:** 5-HIAA urine (Cutoff 5-HIAA urine 3 (ng/moles of creatinine)).

Diagnostic Characteristics	Value	95% CI
Sensitivity	79.31%	60.28% to 92.01%
Specificity	80.00%	28.36% to 99.49%
Positive Likelihood Ratio	3.97	0.68 to 23.12
Negative Likelihood Ratio	0.26	0.11 to 0.60
Positive Predictive Value	95.83%	79.78% to 99.26%
Negative Predictive Value	40.00%	22.41% to 60.61%

**Table 5:** 5-HIAA platelet/plasma (Cutoff 5-HIAA platelet/plasma 1.2).

Diagnostic Characteristics	Value	95% CI
Sensitivity	67.39%	51.98% to 80.47%
Specificity	80.00%	59.30% to 93.17%
Positive Likelihood Ratio	3.37	1.50 to 7.57
Negative Likelihood Ratio	0.41	0.26 to 0.65
Positive Predictive Value	86.11%	73.40% to 93.30%
Negative Predictive Value	57.14%	45.72% to 67.85%
Accuracy	71.83%	59.90% to 81.87%

**Table 6:** 5-HIAA plasma/urine (Cutoff 5-HIAA plasma/urine 3.5).

Diagnostic Characteristics	Value	95% CI
Sensitivity	75.86%	56.46% to 89.70%
Specificity	80.00%	28.36% to 99.49%
Positive Likelihood Ratio	3.79	0.65 to 22.16
Negative Likelihood Ratio	0.30	0.14 to 0.66
Positive Predictive Value	95.65%	79.02% to 99.23%
Negative Predictive Value	36.36%	20.76% to 55.49%
Accuracy	76.47%	58.83% to 89.25%

**Table 7:** IGF-1 in plasma (Cutoff 11 ng/mL).

Diagnostic Characteristics	Value	95% CI
Sensitivity	54.35%	39.01% to 69.10%
Specificity	92.00%	73.97% to 99.02%
Positive Likelihood Ratio	6.79	1.75 to 26.35
Negative Likelihood Ratio	0.50	0.35 to 0.69
Positive Predictive Value	92.59%	76.32% to 97.98%
Negative Predictive Value	52.27%	43.91% to 60.51%
Accuracy	67.61%	55.45% to 78.24%

**Table 8:** IGF-1 index (plasma/platelet) Cutoff 0.65.

Diagnostic Characteristics	Value	95% CI
Sensitivity	95.00%	75.13% to 99.87%
Specificity	85.71%	42.13% to 99.64%
Positive Likelihood Ratio	6.65	1.08 to 40.94
Negative Likelihood Ratio	0.06	0.01 to 0.40
Positive Predictive Value	95.00%	75.53% to 99.15%
Negative Predictive Value	85.71%	46.45% to 97.65%
Accuracy	92.59%	75.71% to 99.09%

## Data Availability

The data that support the findings of this study are available upon request from the corresponding author due to privacy reason.
